# Striatal and Hippocampal Atrophy in Idiopathic Parkinson’s Disease Patients without Dementia: A Morphometric Analysis

**DOI:** 10.3389/fneur.2017.00139

**Published:** 2017-04-13

**Authors:** Jared J. Tanner, Nikolaus R. McFarland, Catherine C. Price

**Affiliations:** ^1^Clinical and Health Psychology, University of Florida, Gainesville, FL, USA; ^2^Neurology, University of Florida, Gainesville, FL, USA; ^3^Center for Movement Disorders and Neurorestoration, University of Florida, Gainesville, FL, USA

**Keywords:** Parkinson’s disease, morphometry, neostriatum, magnetic resonance imaging, hippocampus

## Abstract

**Background:**

Analyses of subcortical gray structure volumes in non-demented idiopathic Parkinson’s disease (PD) often, but not always, show volume loss of the putamen, caudate nucleus, nucleus accumbens, and hippocampus. There is building evidence that structure morphometry might be more sensitive to disease-related processes than volume.

**Objective:**

To assess morphometric differences of subcortical structures (putamen, caudate nucleus, thalamus, globus pallidus, nucleus accumbens, and amygdala) as well as the hippocampus in non-demented individuals with PD relative to age and education matched non-PD peers.

**Methods:**

Prospective recruitment of idiopathic no-dementia PD and non-PD peers as part of a federally funded investigation. T1-weighted isovoxel metrics acquired *via* 3-T Siemens Verio for all individuals [PD *n* = 72 (left side onset *n* = 27, right side onset *n* = 45); non-PD *n* = 48]. FIRST (FMRIB Software Library) applications provided volumetric and vertex analyses on group differences for structure size and morphometry.

**Results:**

Group volume differences were observed only for putamen and hippocampi (PD < non-PD) with hippocampal volume significantly associating with disease duration. Group shape differences were observed for bilateral putamen, caudate nucleus, and hippocampus with greater striatal atrophy contralateral to side of motor symptom onset. Hippocampal shape differences disappeared when removing the effects of volume.

**Conclusion:**

The putamen was the primary structure to show both volume and shape differences in PD, indicating that the putamen is the predominant site of basal ganglia atrophy in early- to mid-stage PD. Side of PD symptom onset associates with contralateral striatal atrophy. Left-onset PD might experience more extensive striatal atrophy than right-onset PD. Hippocampus morphometric results suggest possible primary atrophy of CA3/4 and dentate gyrus.

## Introduction

Analyses of subcortical gray structure volumes in non-demented idiopathic Parkinson’s disease (PD) show volume loss of the putamen (1–3), caudate nucleus (4, 5), nucleus accumbens (3), and hippocampus (6–8). Not all studies, however, show volume differences in non-demented PD relative to non-PD peers (9, 10). These findings likely indicate heterogeneity of disease progression and suggest that volume is not an optimal biomarker for PD pathology.

There is growing evidence that morphometric analyses are more sensitive to subcortical structural changes associated with PD than volumetrics alone. To date, there have been 10 studies assessing subcortical shape in PD (Table 1). Patterns of subcortical change (including atrophy and hypertrophy) in PD are emerging with the putamen (3, 11–14) and caudate nucleus (11, 13–15) most consistently demonstrating local atrophy. Thalamus (13, 16), globus pallidus (13, 17), and nucleus accumbens (18) differences have also been shown. Not every study, however, included analyses of the same structures.

**Table 1:** **Summary of research covering subcortical shape analyses in Parkinson’s disease (PD)**.

Reference	*N*	PD duration (years)	Hoehn and Yahr	Shape method	Structures analyzed	Regions of shape significance
McKeown et al. (16)	18 PD:18 Non-PD	3.6 ± 2.6	2–3	SPHARM	Thal	Left and right thalamus
Apostolova et al. (15)	12 PDND, 8 PDMCI, 15 PDD: 20 non-PD	14.3 ± 5.1 PDND, 10.5 ± 4.3 PDMCI, 13.1 ± 7.8 PDD	2–3	Radial distance mapping	Hipp, Caud, Lat Vent	Left and right caudate head in PDMCI and PDD versus non-PD; PDD larger ventricles bilaterally versus non-PD and larger right versus PDND and PDMCI
Sterling et al. (11)	40 PD: 40 non-PD	4.1 ± 4.2	1.8 ± 0.6	SPHARM-PDM	Puta and Caud	Putamen and caudate; putamen atrophy greater contralateral than ipsilateral
Lee et al. (3)	49 PD: 53 non-PD	1.37 ± 1.21	2.0 ± 0.5	FSL FIRST vertex	Thal, Caud, Puta, GP, Nuc Accu, Hipp, Amyg	Left and right posterolateral and ventromedial putamen
Mak et al. (21)	65 PD-NCI: 25 PD-MCI	5.4 ± 4.3 PD-NCI, 5.0 ± 2.7 PD-MCI	1.88 ± 0.39	FSL FIRST vertex	Thal, Caud, Puta, GP, Nuc Accu, Hipp, Amyg	No PD-NCI and PD-MCI group differences
Menke et al. (17)	20 PD: 19 non-PD	1.8 ± 0.8	1.8 ± 0.4	FSL FIRST vertex	Thal, Caud, Puta, GP, Nuc Accu, Hipp, Amyg	Right GP
Garg et al. (13)	244 PD: 191 non-PD	Not specified	Not specified	Surface displacement based on LDDMM	Thal, Caud, Puta, GP	Bilateral thalamus, caudate, putamen, GP
Nemmi et al. (14)	21 PD: 20 non-PD	7.4 ± 5.4	2.0 ± 0.2	FSL FIRST vertex	Thal, Caud, Puta, GP, Nuc Accu, Hipp, Amyg	Left caudate, bilateral putamen
Nyberg et al. (18)	21 PD: 20 non-PD	5.5 ± 3.4	2.2 ± 0.7	FSL FIRST vertex	Thal, Caud, Puta, GP, Nuc Accu, Hipp, Amyg	Right nucleus accumbens
Caligiuri et al. (12)	42 PD: 30 non-PD	1.33 ± 0.8	1.5 ± 0.6	FSL FIRST vertex	Thal, Caud, Puta, GP, Nuc Accu, Hipp, Amyg	Bilateral putamen

*SPHARM, spherical harmonics; SPHARM-PDM, spherical harmonics point distribution model; LDDMM, large deformation diffeomorphic metric mapping; PDND, Parkinson’s disease (PD) non-demented; PDMCI, PD mild cognitive impairment; PDD, PD dementia; PD-NCI, PD not cognitively impaired; PD-MCI, PD mild cognitive impairment; Thal, thalamus; Caud, caudate nucleus; Puta, putamen; GP, globus pallidus; Nuc Accu, nucleus accumbens; Hipp, hippocampus; Amyg, amygdala*.

Two studies (11, 12) additionally addressed symptom laterality and lateralized atrophy with the suggestion that there were greater contralateral than ipsilateral shape changes in PD, but the results could benefit from replication with larger samples. An additional limitation of previous studies is that while atrophy of the hippocampus and related medial temporal lobe cortex are reported as common features in PD (7, 8, 19, 20), morphometric studies have either not addressed (11, 13, 16, 17) or found (3, 12, 14, 15, 18, 21) hippocampal shape differences.

An additional consideration is the relationship between volumetric representation and side of onset. There is evidence of greater contralateral neuronal loss in the substantia nigra in PD (22) but findings with other neuroanatomical structures are mixed. Lewis and colleagues (23) demonstrated lateral ventricular volume asymmetry (contralateral to symptom laterality) in PD but lateral ventricles are only indirect measures of atrophy and the results have not been replicated by others (24). Basal ganglia neuronal loss also typically appears to be bilateral, regardless of side of onset (2). The idea of volumetric asymmetry (contralateral < ipsilateral) in PD is thus a tentative hypothesis. Assessing gross structural differences such as volume, however, likely obscures subtle within structure changes.

For these reasons, we designed the current study to assess differences in volumetrics and shape in the putamen, caudate nucleus, thalamus, globus pallidus, nucleus accumbens, amygdala, and hippocampus in a well-characterized group of non-demented individuals with PD compared to a closely matched group of peers without PD. The amygdala was included given previous research demonstrating pathology and atrophy of the structure in PD (25); it was also included because of its close connections to the hippocampus and to apathy, which is prevalent in PD (26). We hypothesized group morphometric differences (i.e., atrophy in PD relative to non-PD) within striatal and hippocampal structures with striatal atrophy most prevalent. We also addressed side of onset as it relates to lateralized subcortical shape differences, with the hypothesis that greater volumetric and shape difference would occur contralateral to side of onset.

## Participants and Methods

### Participants

Participants were recruited from an ongoing prospective federally funded investigation.

Inclusion criteria for PD participants included diagnosis by a movement disorder neurologist using the UK Parkinson’s Disease Society Brain Bank Clinical Diagnostic criteria (27), and scores of 1–3 on the Hoehn and Yahr Scale. Inclusion criteria for all participants included ≥age 60, at least a fifth grade reading level, right-handedness, absence of dementia based on scores on two screening measures [modified telephone interview of cognitive status score > 34 (28), dementia rating scale-2 raw score > 130 (29)], and English as a first language. Exclusion criteria included secondary/atypical parkinsonism, deep brain stimulation surgery, major psychiatric disorders, and vascular diseases most likely to confound cognition (e.g., cerebrovascular accident within the past 6 months, congestive heart failure, etc.), and axial/gait symptoms as initial PD symptom. Recruitment efforts for PD participants involved (1) direct neurology referrals from the UF Center for Movement Disorders and Neurorestoration (UF CMDNR), (2) identification of individuals from the UF CMDNR’s cognitive research database (*n* > 600), and (3) advertisement at community PD support groups. Non-PD control participants were recruited from the UF Age Network Registry, family members of PD participants, community fliers, and free community memory screenings. Study participants were Caucasian, generally well-educated, and lived in northern Florida.

At the time of this analysis, 126 individuals were enrolled in the larger parent study. Data from 72 individuals with PD and 48 non-PD peers with artifact-free MRI and complete demographic and general cognitive measures were included in the current investigation. On medication, UPDRS scores were acquired at time of testing to represent typical participant function. Side of onset was based on participant self-report and medical record review (left side onset = L-PD, *n* = 27; right side onset = R-PD, *n* = 45). All included PD participants had tremor dominant onset. Data from a subset of these participants have been discussed in previous reports (20, 30, 31).

### MRI Acquisition and Processing

We acquired MRI data using a Siemens 3-T Verio scanner with an 8-channel head coil. As a subset of a larger imaging protocol, we acquired a T1-weighted sequence with the following parameters: 176 contiguous slices, 1 mm^3^ voxels, TR/TE = 2,500/3.77 ms.

#### Subcortical Structure Segmentation

We converted all T1 raw dicom images to Nifti format using *dcm2niix* (https://www.nitrc.org/projects/mricrogl/). To reduce segmentation errors, we ran FSL’s *robustfov* to remove neck before volumetric and shape processing. We verified that each T1 included all portions of the head superior to the foramen magnum. Using FIRST (32), we segmented bilateral amygdala, caudate nucleus, globus pallidus, hippocampus, nucleus accumbens, putamen, and thalamus. The segmentation by FIRST is achieved by registering a template model to the individual T1 image to be segmented. The probability of the shape, based on the observed intensity, is then calculated. FIRST creates a surface mesh for each subcortical structure using a deformable mesh model. Each mesh is made up of a set of triangles with the apex of connecting triangles called a vertex. FIRST sets the number of vertices for each structure to be equal in order for the corresponding vertices to be compared across individuals. The meshes are then converted to 3D volumes using a boundary correction that defines each voxel as being inside or outside the structure. Visual inspection (Jared J. Tanner) of all segmentations revealed no failure.

#### Volumetric Analysis

We calculated volumes for each structure using *fslstats*. To adjust for head size differences, we divided the raw volumes by the total intracranial volume (TICV). We estimated TICV by adding all CSF, gray, and white matter voxels [brainmask.mgz created by FreeSurfer (33, 34)]. This estimation of TICV compared to the gold standard of manual segmentation is both reliable and robust [dice similarity coefficient = 0.95, ICC = 0.92, *n* = 80 (35)]. The final variable of interest was the ratio of subcortical volume to TICV.

#### Shape Analysis

Although each vertex represents the same point in space for different subjects for each structure, the surfaces are in native image spaces. Before any group level analysis can be performed, vertices are registered into a common space—in this instance, the mean surface of the sample represented in MNI152 space. For each participant, rotation and translation (pose) are removed by minimizing the sum-of-squares differences between the corresponding vertices of the individual and mean surfaces. We used a six-degree of freedom (DOF) transformation to remove pose. The six DOF registration removes only translations and rotations of a rigid body transformation so that differences in both volume and shape are retained [we refer to this as a *shape considering volume* analysis (32)]. We also repeated the analyses adjusting the models for scale (*first_utils—useScale*) in order to limit changes just to shape, rather than shape and volume (we refer to this as a *shape only* analysis). To correct for head size differences, meshes were reconstructed in MNI space, which is the native model space.

#### Statistical Analyses

Subcortical volumes as a ratio to TICV were compared between groups (PD versus non-PD and left-onset PD versus right-onset PD versus non-PD) using MANCOVA in SPSS 22.0 (IBM, New York) with significance set at *p* < 0.05. We covaried for both age and sex. Total volumes were the sum of left and right structures.

We performed both *shape considering volume* and *shape only* morphometric difference analyses between all PD and non-PD peers, left-onset PD versus right-onset PD, left-onset PD versus non-PD peers, and right-onset PD versus non-PD peers. The significance of these tests was calculated using a Monte Carlo simulation together with a threshold-free cluster enhancement as implemented in FSL *randomise* (35). The groups were well matched on demographic variables in PD/non-PD between-group statistical analyses. *T*-values obtained in the non-permuted data were compared against a null distribution calculated using 10,000 random permutations of the data. We then thresholded images at *p* < 0.05, corrected for multiple comparisons, for display. For participants with PD, we performed additional morphometric analyses correlating *shape considering volume* and *shape only* with disease duration in years while covarying for age.

## Results

### Participants

Demographic information is displayed in Table 2. Groups were well matched for all variables not related to disease (all *p*-values > 0.42).

**Table 2:** **Demographic information**.

	All PD (*n* = 72)	Left-onset PD (*n* = 27)	Right-onset PD (*n* = 45)	Non-PD (*n* = 48)
Age	67.75 ± 5.86	67.19 ± 5.01	68.09 ± 6.34	68.17 ± 4.97
Education	16.28 ± 2.67	16.33 ± 2.29	16.24 ± 2.89	16.89 ± 2.24
Sex (M:F)	51:21	19:8	32:13	38:10
Disease duration	7.00 ± 4.98	5.37 ± 3.10**	7.98 ± 5.63**	–
MMSE	28.59 ± 1.33*	28.59 ± 1.34	28.58 ± 1.31	29.26 ± 0.94*
WTAR estimated IQ	109.46 ± 7.34	109.52 ± 6.92	109.40 ± 7.76	109.64 ± 8.41
UPDRS part 3	18.04 ± 10.09*	17.74 ± 8.83	18.28 ± 10.92	2.96 ± 3.57*
LED	585.07 ± 334.34*	539.00 ± 232.69	631.13 ± 435.99	0.85 ± 5.83*^,a^

*UPDRS part 3 is the motor symptom scale of the United PD Rating Scale; LED, Levodopa equivalence score; MMSE, mini-mental state examination; WTAR, Wechsler Test of Adult Reading*.

*^a^One non-PD participant was taking levodopa for restless leg syndrome*.

*Indicates significant PD/non-PD difference, p < 0.05.

**Indicates significant L-PD/R-PD difference, p < 0.05.

### Volume Analyses: Amygdala, Caudate Nucleus, Globus Pallidus, Hippocampus, Nucleus Accumbens, Putamen, and Thalamus

The one-way between-group (PD versus non-PD) multivariate analysis demonstrated that volumes for subcortical nuclei in PD subjects were smaller (*F* = 4.34, *p* < 0.001; raw volumes are presented in Table S1 in Supplementary Material). Univariate differences were significant for only total putamen (*F* = 12.204, *p* = 0.001) and total hippocampal (Pillai’s trace *F* = 11.894, *p* = 0.001) volumes, with a trend for total caudate nucleus (*F* = 3.685, *p* = 0.057). Globus pallidus, thalamus, nucleus accumbens, and amygdala volumes were not different between groups (all *p*-values > 0.391).

When groups with left and right side PD onset were analyzed separately, a one-way between-group (L-PD, R-PD, non-PD) MANCOVA with total caudate, putamen, nucleus accumbens, thalamus, globus pallidus, hippocampus, and amygdala volume (adjusted for TICV) with age and sex as covariates showed a significant overall group effect (Pillai’s trace *F* = 2.433, *p* = 0.003; Bonferroni correction applied). Univariate (pairwise) comparisons controlling for age and sex demonstrated significant group differences in putamen (*F* = 6.126, *p* = 0.003), caudate nucleus (*F* = 3.555, *p* = 0.032), and hippocampus (*F* = 5.99, *p* = 0.003) volumes. No other structures were different (all *p*-values > 0.49).

Both L-PD and R-PD had smaller total putamen (L-PD versus non-PD: *p* = 0.012; R-PD versus non-PD: *p* = 0.011) volume than non-PD. R-PD had smaller total hippocampus volume than non-PD (*p* = 0.004) with L-PD at trend significance (*p* = 0.053). Only L-PD had smaller total caudate nucleus volumes than non-PD (*p* = 0.027; R-PD versus non-PD *p* > 0.54). L-PD and R-PD did not differ for any total structure volumes (all *p*-values > 0.20).

**Figure 1:**
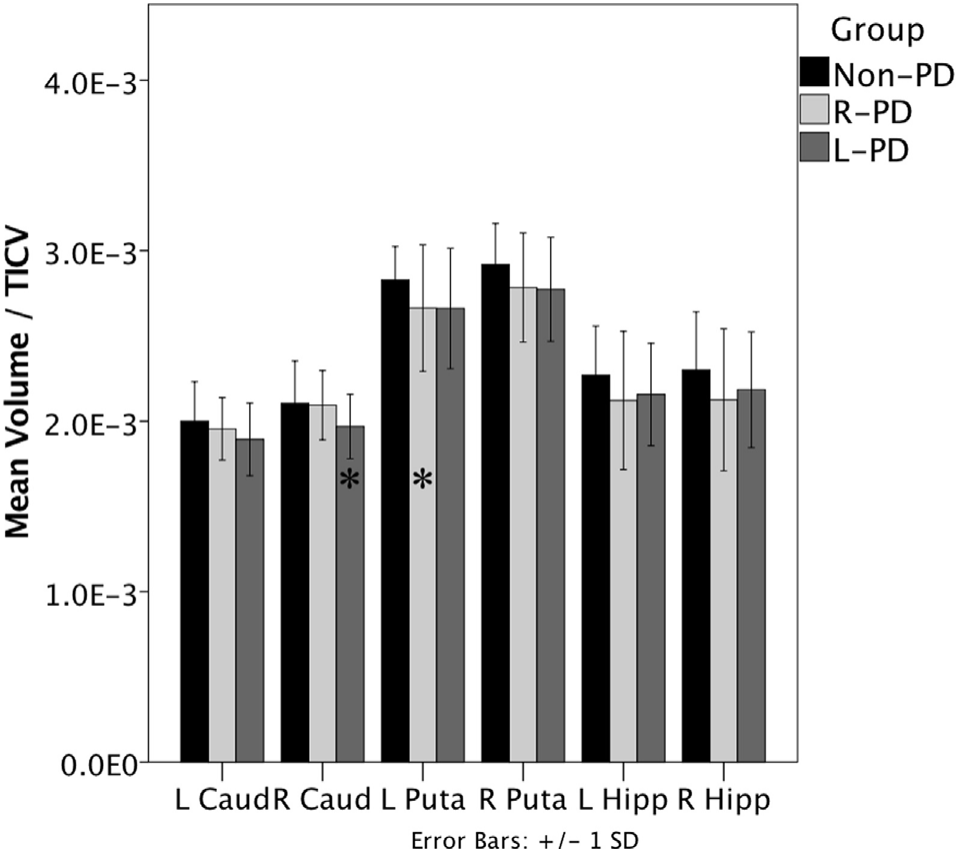
**Right-onset Parkinson’s disease (PD) (*n* = 45) and left-onset PD (*n* = 27) versus non-PD (*n* = 48) volumetric differences**. Note: all values are the group mean ratios of structure volume relative to total intracranial volume (TICV). Error bars indicate ±1 SD. *Indicates L-PD or R-PD < non-PD, *p* ≤ 0.05.

When assessing structure volume by hemisphere, L-PD had smaller right caudate nucleus volumes than non-PD (*p* = 0.038; see Figure 1 and Figure S1 in Supplementary Material) and trend significance for left putamen (*p* = 0.080). R-PD had smaller left putamen volumes than non-PD (*p* = 0.035; see Figure 1) and trend significance for right putamen (*p* = 0.072) and right hippocampus (*p* = 0.074). L-PD and R-PD did not differ significantly for any structure volume (all *p*-values > 0.066).

Hippocampus volume negatively associated with disease duration when controlling for age (partial *r* = −0.299, *p* = 0.013). The hippocampus/disease duration relationship was significant only for R-PD (partial *r* = −0.315, *p* = 0.042; L-PD partial *r* = −0.263, *p* = 0.195). Putamen volume showed a trend relative to disease duration (partial *r* = −0.218, *p* = 0.073).

### Shape Analyses: Amygdala, Caudate Nucleus, Globus Pallidus, Hippocampus, Nucleus Accumbens, Putamen, and Thalamus

**Figure 2:**
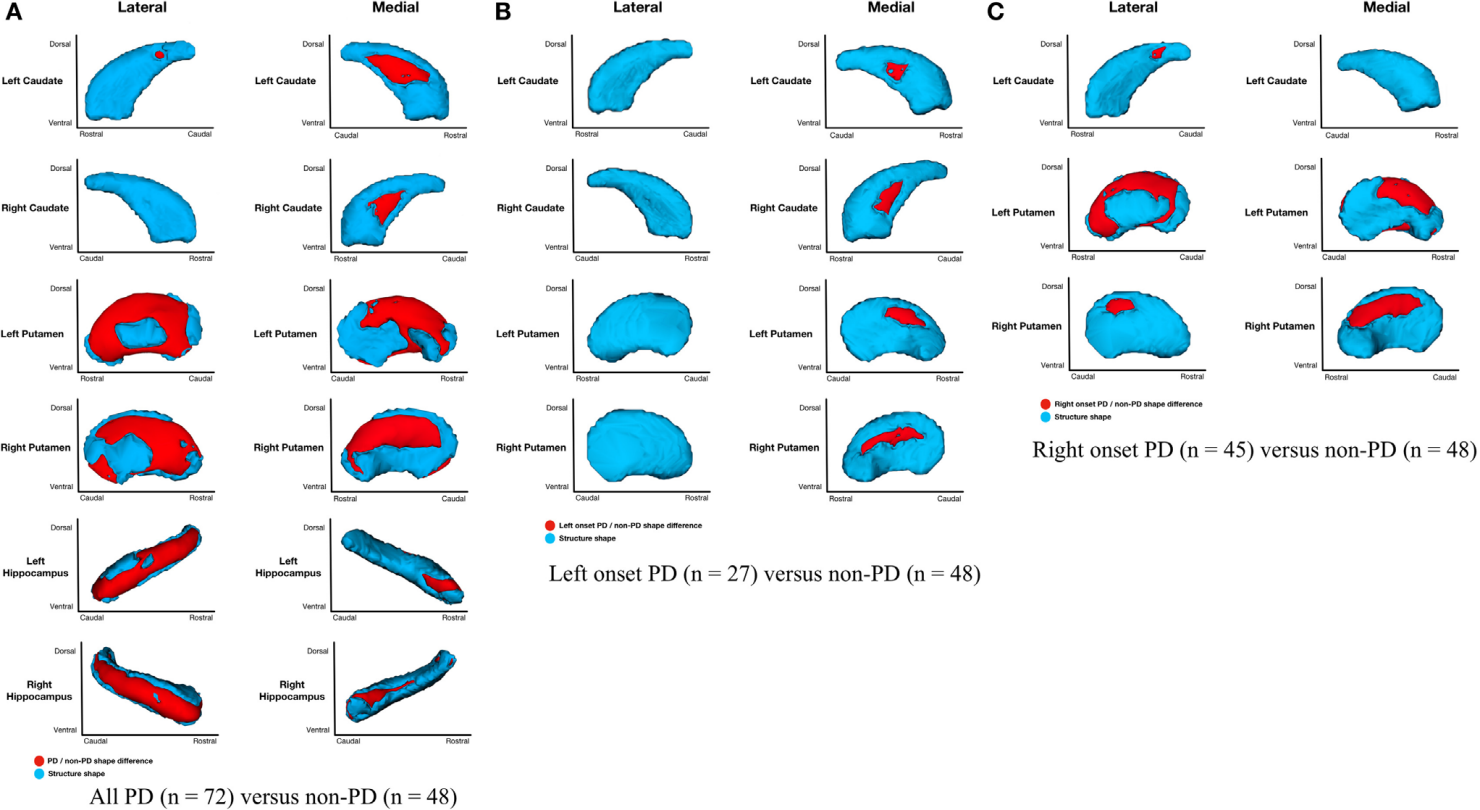
**Parkinson’s disease (PD) and non-PD subcortical *shape considering volume* differences**. Note: areas in red indicate where groups are significantly different (FDR-corrected *p* < 0.05). Areas in red indicate significant *F-*values, which in this case are all areas of atrophy (inward vertex displacement) in PD relative to non-PD peers. Relative to non-PD, L-PD have more right than left putamen and caudate nucleus atrophy and R-PD have more left than right putamen and caudate nucleus atrophy (see Table 3). **(A)** All PD (*n* = 72) versus non-PD (*n* = 48); **(B)** left onset PD (*n* = 27) versus non-PD (*n* = 48); **(C)** right onset PD (*n* = 45) versus non-PD (*n* = 48).

Parkinson’s disease (*n* = 72) relative to non-PD (*n* = 48) had *shape considering volume* differences on the medial and lateral surfaces of left and right putamen (primarily dorsal), posteriomedial, and posteriolateral surfaces of the left caudate nucleus, medial surface of right caudate nucleus, and medial and lateral regions of left and right hippocampi (Figure 2A). All areas of significant difference represented atrophy in PD relative to non-PD (i.e., there were no areas of hypertrophy). No other structures showed differences.

**Figure 3:**
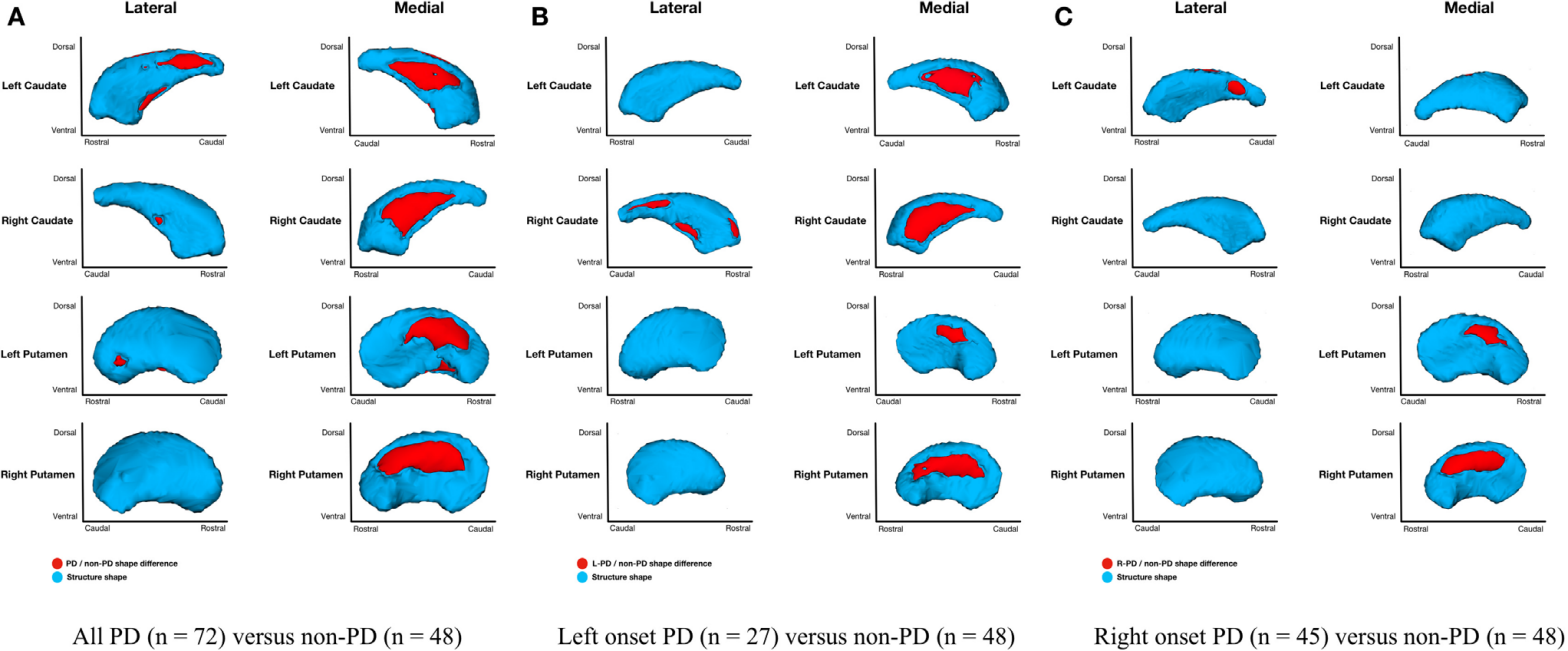
**Parkinson’s disease (PD) and non-PD subcortical *shape only* differences**. Note: areas in red indicate where groups are significantly different (FDR-corrected *p* < 0.05). Areas in red indicate significant *F*-values, which in this case are all areas of atrophy (inward vertex displacement) in PD relative to non-PD peers. Relative to non-PD, L-PD have more right than left putamen and caudate nucleus atrophy and R-PD have more left than right caudate nucleus atrophy but more right than left putamen atrophy (see Table 3). **(A)** All PD (*n* = 72) versus non-PD (*n* = 48); **(B)** left onset PD (*n* = 27) versus non-PD (*n* = 48); **(C)** right onset PD (*n* = 45) versus non-PD (*n* = 48).

Parkinson’s disease (*n* = 72) relative to non-PD (*n* = 48) had *shape only* (adjusted for structure scale, which removes the effects of volume) differences for bilateral putamen and caudate nuclei. Group differences were more extensive medially than laterally with more differences anterior and dorsal (Figure 3A). Without considering volume, there were no left or right hippocampal group differences. Disease duration within PD group and regardless of group did not significantly correlate with putamen, caudate nucleus, or hippocampus morphometry.

#### Left-Onset PD Relative to Non-PD

*Shape considering volume* differences in left PD onset (*n* = 27) relative to non-PD peers (*n* = 48) were significant only for bilateral medial putamen and bilateral medial caudate nucleus (Figure 2B). In the *shape only* analysis, there were medial differences for bilateral putamen and caudate nuclei. There were also lateral right caudate nucleus shape differences (Figure 3B).

#### Right-Onset PD Relative to Non-PD

*Shape considering volume* differences in right PD onset (*n* = 45) relative to non-PD peers (*n* = 48) were significant for medial and lateral putamen bilaterally (more extensive on the left) and left posteriolateral caudate nucleus (Figure 2C). Differences in the putamen tended to be dorsal and anterior in the structure. There were no right caudate nucleus group differences. In a *shape only* analysis, there were medial putamen differences bilaterally (more extensive on the right) and left posteriolateral caudate nucleus differences (Figure 3C).

#### Left-Onset PD Relative to Right-Onset PD

Left-onset PD (*n* = 27) versus right-onset PD (*n* = 45): there were no *shape considering volume* differences between L-PD and R-PD. A *post hoc* analysis covarying for disease duration also showed no between-group differences for any structure.

## Discussion

This study revealed that the putamen, caudate nucleus, and hippocampus have unique volume and shape profiles in non-dementia PD. The putamen was the only structure to show differences in *both* volume and shape. While finding putamen atrophy in PD is not novel (3, 11–14), our analysis extends the understanding of putamen morphology relative to the caudate nucleus and hippocampus in PD. The caudate nucleus, in contrast to the putamen, had only shape differences on the lateral surface and was more pronounced contralateral to side of onset. The hippocampus, the only cortical structure of interest, showed a significant volumetric difference relative to non-PD peers (5.96% less), yet no external shape difference. These findings suggest that volume loss is driven by internal structural differences (e.g., dentate gyrus) rather than the external shape morphology that occurs for the putamen and caudate nuclei.

### Striatal Patterns

The putamen and caudate show medial and lateral differences—but the extent of these differences varies when we correct for structure volume. For PD, the putamen’s primary morphmetric difference was on the medial surface, whereas the caudate nuclei had both clear lateral and medial atrophy.

When assessing volume only, there were no clear patterns of structure asymmetry relative to side of motor onset (see Table 3). In contrast, while shape differences were evident bilaterally for both L-PD and R-PD relative to non-PD peers, contralateral putamen or caudate nuclei generally showed larger areas of atrophy (see Table 3). This result thus matches previous morphometric research (11, 12) and demonstrates the limitations of assessing asymmetry using traditional volumetrics (2). That there were also ipsilateral shape differences was expected because of the mean disease duration and manifestation of bilateral clinical symptoms. We also found that L-PD had extensive caudate nucleus morphometric and volume differences relative to non-PD peers. In contrast, the caudate nucleus was less affected in R-PD. Our result provides tentative evidence of increased susceptibility for caudate nucleus atrophy with left-onset PD relative to R-PD.

**Table 3:** **Ratios of left and right structures relative to non-PD peers**.

		Structure	L-PD	R-PD	All PD
Volume only		Putamen	1.17	1.24	1.22
		Caudate nucleus	0.83	0.32	1.27
		Hippocampus	0.99	0.86	0.90
Shape considering volume		Putamen	1.36	0.39	0.68
		Caudate nucleus	1.71	0.00	0.44
		Hippocampus	–	–	0.54
Shape only		Putamen	2.84	2.30	0.84
		Caudate nucleus	2.23	0.00	0.91
		Hippocampus	–	–	–

	More left structure atrophy relative to right				
	More right structure atrophy relative to left				

*All values >1 indicate more right side atrophy whereas all values <1 indicate more left side atrophy. For volume only, the value was calculated as the PD mean volume difference from non-PD mean volume as a ratio of mean non-PD volume (left mean non-PD structure volume—left mean PD structure volume/left mean non-PD volume) to (right mean non-PD structure volume —right mean PD structure volume/right mean non-PD volume); the ratio is thus left/right where a value >1 = smaller right structure than left and value <1 = smaller left than right. For both shape considering volume and shape only, the value was calculated as the ratio of [right number of significantly different voxels (PD relative to non-PD)/total structure voxels] to [left number of significantly different voxels (PD relative to non-PD)/total structure voxels]; the ratio is thus right/left where a value >1 = more atrophy on the right than left and <1 = more left atrophy than right. – indicates no group difference and thus no way to assess asymmetry*.

Taken together, our results demonstrate greater contralateral striatal atrophy relative to side of motor onset in PD and a medial-to-lateral progression of atrophy within the striatum (12). An alternative interpretation is that the medial sides of the putamen and caudate nucleus might be the sides of the structures that shift with neurodegeneration. In other words, the lateral surfaces might be relatively “fixed” in place so atrophy anywhere within the striatal structures shifts the medial surface.

Striatal atrophy can be expected given the location and spread of PD pathology. Alpha-synuclein aggregation and/or dopaminergic (DA) denervation, however, do not necessarily indicate atrophy. DA neurons in the substantial nigra pars compacta (SNc) and ventral tegmental area (VTA) of the midbrain undergo significant degeneration in PD, although the VTA to a lesser extent than the SNc, because of PD pathology. Both regions project directly to the striatum and other subcortical gray matter structures *via* the mesostriatal pathway (36). While the striatum exhibits a posterior/anterior gradient of striatal dopamine loss in PD (37, 38), our results do not clearly demonstrate a posterior > anterior atrophy pattern. The lack of this pattern indicates that atrophy of the striatum is not a structural MRI marker of DA denervation.

Importantly, our results also suggest more extensive striatal atrophy contralateral to side of motor symptom onset, bolstering the findings of previous research (11, 12, 23). This is particularly evident for the lateral (away from lateral ventricles) side of the contralateral caudate nucleus.

Progression of striatal pathology has implications for brain networks. The striatum connects broadly to many areas of the cortex (39). An fMRI connectivity analysis showed that blood-oxygen-level-dependent (BOLD) activity in the medial portion of the putamen (regions where the *shape only* analysis primarily showed group differences) is associated with BOLD signal in the SMA with possible DLPFC and amygdala involvement. Similarly, affected regions of the caudate nuclei possibly connect to calcarine, inferior temporal, insula, superior temporal, cingulate, medial prefrontal, orbitofrontal, and medial temporal regions (40).

The loss of gray matter within the striatum has broad consequences for cognition, particularly, speed of processing, which is the primary cognitive deficit of early-stage PD (30). It is still not clear, however, exactly how PD pathology, atrophy, and clinical symptoms interact. There are individuals who have widespread PD pathology at death but who did not exhibit symptoms of PD, which is part of the controversy regarding the spread of pathology in PD (41–47). In order to track pathology progression more reliably and predict clinical symptoms, detecting *in vivo* markers of PD pathology spread is necessary. PET and SPECT imaging are promising methods to detect spread of pathology but are hindered by intra- and inter-individual variability [for a brief review, refer to Ref. (48)]. MRI-based imaging markers of PD pathology spread are less clear, but no less important, for understanding the asymmetry and/or symmetry of progression of PD brain and clinical changes.

Previous research finding no asymmetry of structural changes in PD were limited by less sensitive metrics (i.e., volume), leading to the conclusion that side of onset does not affect gross gray matter structure (2). Whole structure volume is important but has the potential to mask subtle differences and changes. Using more sensitive MRI metrics, a number of studies including this one have started to show contralateral structure changes in PD (49, 50). Asymmetric structural alterations associated with PD are thus becoming clear. A caveat is that functional deficits become less lateralized as PD progresses (38), so it is also possible that detection of asymmetric atrophy becomes more difficult as the disease progresses. Longitudinal data are necessary to clarify this hypothesis. However, structural asymmetry, as the quality of MRI data and sensitivity of analyses increase, has the potential to be considered along with other proposed markers as useful for the detection of prodromal PD (51).

### Hippocampal Patterns

While the striatum appears to be the primary site of atrophy within PD, we found the hippocampus was the only structure where volume associated with disease duration, even when controlling for age and UPDRS part 3 score. We conclude that areas within the temporal lobe might serve as biomarkers of disease progression in PD, although such a conclusion is tentative without longitudinal data.

There were extensive bilateral hippocampal shape differences when including effects of volume. This pattern was expected given the smaller hippocampal volumes in PD, which has been shown in other studies (7, 52) in addition to ours. When including volume effects, hippocampal differences appeared to affect mainly the hippocampal head and CA1 bilaterally with less extensive CA2/3 atrophy. However, because the PD/non-PD hippocampus group differences disappeared when removing effects of volume (i.e., focusing on external shell of the structure independent from internal volume), we speculate that changes with PD are involving CA2/3 and dentate rather than CA1. This interpretation is supported by previous research, which showed that non-demented individuals with PD have smaller CA2/3 and dentate gyrus volumes relative to non-PD peers with the rest of hippocampus subregions (including CA1) remaining relatively intact early in the disease process (53). Loss of gray matter in CA3 and dentate gyrus has implications for white matter connectivity important for memory in light of atrophy of entorhinal cortex in PD (8, 20) and the white matter (e.g., perforant pathway) connecting both regions. This remains an area requiring further research.

While there are significant between-group volume differences, our findings in conjunction with Pereira et al. (53) demonstrate the local nature of hippocampal changes associated with PD. That is, extensive portions of the hippocampus are relatively unaffected by cell loss in early- to mid-stage PD. Therefore, assessing whole structure volumes, even with significant differences, hides important localized changes, which might be apparent earlier in the disease process. This is important because patients with PD who have hippocampal atrophy relative to peers are at increased risk of future dementia (19). There is a need to associate cognitive ability and change over time, as there are subgroups of PD with amnestic mild cognitive impairment profiles (20).

We recognize that the current research is limited by statistical power to examine side of onset differences particularly in the left side onset sample. Further, side of onset is based on self-report of first symptoms and is limited by patient recall. The results could be improved by using a more direct measure of disease severity and laterality (e.g., off medication UPDRS); those data were not available, so we opted for disease duration as an indirect marker of disease severity. Additionally, we recognize that the testing was conducted while on-medication to represent typical patient functioning, which limits information about the extent of disease severity. We also recognize the disease duration differences between the L-PD and R-PD groups. The groups, however, did not have significant shape differences or shape/disease duration correlations, which indicates that the disease duration differences did not have a detectable effect on our analyses. We also note that the asymmetry results presented in Table 3 are qualitative as no formal statistical tests were performed to assess right/left differences. All asymmetrical atrophy results are thus preliminary and tentative until further research is conducted. We encourage future researchers to conduct research replicating our findings but also to examine structural profiles relative to cognitive performance and, in particular, performance over time and dementia prediction.

Despite these limitations, the current study provides compelling data showing that the striatum is a primary site of cell loss in early- to mid-stage PD; the putamen and caudate primarily have medial shape changes with this dominantly contralateral to symptom onset. Individuals with left-onset symptoms also appear to have more extensive striatal atrophy, which finding needs to be investigated further. Additionally, PD associates with hippocampal volumetric loss (not shape) suggesting greater demise within the hippocampus (e.g., dentate gyrus) as the disease progresses. Future studies are encouraged to conduct shape and volume assessments relative to cognitive and memory performances in PD.

## Ethics Statement

The study was approved by the University of Florida Health Center Institutional Review Board (Protocol #472–2007). The research was conducted in accordance with the recommendations of the University of Florida Health Center Institutional Review Board. Written and informed consent was obtained from all participants and the research followed the Declaration of Helsinki of 1975.

## Author Contributions

JT and CP: conceptualization, acquisition, analysis, drafting, and revisions. NM: interpretation, revisions. All authors (JT, NM, and CP) provided final approval of this manuscript and agreed to be accountable for all aspects of the work.

## Conflict of Interest Statement

The authors declare that the research was conducted in the absence of any commercial or financial relationships that could be construed as a potential conflict of interest.
