# Supplementary material for: Striatal and Hippocampal Atrophy in Idiopathic Parkinson’s Disease Patients without Dementia: A Morphometric Analysis
Source: Front Neurol. 2017 Apr 13;8:139. doi: 10.3389/fneur.2017.00139 (PMC5389981; doi:10.3389/fneur.2017.00139)
Supplement: Supplementary file 1 [file Table_1.DOCX]

Supplementary Material

Striatal and Hippocampal Atrophy in Idiopathic Parkinson Disease Patients without Dementia: A Morphometric Analysis

Jared J. Tanner^1^, Nikolaus R. McFarland^2,3,^, Catherine C. Price^1^

^1^Clinical and Health Psychology, University of Florida, Gainesville, Florida, USA

^2^Neurology, University of Florida, Gainesville, Florida, USA

^3^Center for Movement Disorders and Neurorestoration, University of Florida, Gainesville, Florida, USA

*** Correspondence:**Catherine C. Price, Ph.D., ABBP/CN
cep23@phhp.ufl.edu

# Supplementary Table 1. Native space volumes for Non-PD (n = 48), R-PD (n = 45), and L-PD (n = 27).

| Group | | Minimum | Maximum | Mean | Std. Deviation |
| --- | --- | --- | --- | --- | --- |
| Non-PD | Left Caudate | 2373 | 4501 | 3281.77 | 447.813 |
|  | Right Caudate | 2549 | 4642 | 3450.21 | 457.492 |
|  | Left Putamen | 3843 | 5832 | 4641.63 | 456.896 |
|  | Right Putamen | 3676 | 6022 | 4796.13 | 545.677 |
|  | Left Pallidum | 997 | 2920 | 1689.58 | 325.901 |
|  | Right Pallidum | 1182 | 2422 | 1732.83 | 288.398 |
|  | Left Thalamus | 5586 | 8664 | 7183.42 | 717.759 |
|  | Right Thalamus | 5667 | 8362 | 7065.19 | 625.965 |
|  | Left Accumbens | 213 | 788 | 442.40 | 138.898 |
|  | Right Accumbens | 144 | 612 | 320.46 | 103.254 |
|  | Left Hippocampus | 2635 | 4633 | 3717.13 | 476.226 |
|  | Right Hippocampus | 1823 | 4820 | 3764.38 | 557.596 |
|  | Left Amygdala | 513 | 1708 | 1043.42 | 321.174 |
|  | Right Amygdala | 270 | 1990 | 1099.27 | 390.025 |
| R-PD | Left Caudate | 2547 | 4491 | 3289.73 | 395.307 |
|  | Right Caudate | 2687 | 4340 | 3522.42 | 418.347 |
|  | Left Putamen | 2959 | 6408 | 4475.13 | 643.080 |
|  | Right Putamen | 3535 | 6154 | 4675.40 | 568.397 |
|  | Left Pallidum | 1165 | 3094 | 1815.44 | 380.707 |
|  | Right Pallidum | 1078 | 3812 | 1820.07 | 458.484 |
|  | Left Thalamus | 5122 | 9566 | 7437.62 | 838.558 |
|  | Right Thalamus | 5566 | 9978 | 7316.11 | 727.380 |
|  | Left Accumbens | 206 | 766 | 448.64 | 135.488 |
|  | Right Accumbens | 76 | 600 | 326.42 | 127.082 |
|  | Left Hippocampus | 2166 | 6200 | 3550.89 | 646.087 |
|  | Right Hippocampus | 1508 | 5415 | 3553.98 | 657.720 |
|  | Left Amygdala | 407 | 1894 | 1058.82 | 304.901 |
|  | Right Amygdala | 375 | 2220 | 1211.73 | 360.600 |
| L-PD | Left Caudate | 2287 | 4252 | 3259.70 | 476.477 |
|  | Right Caudate | 2506 | 4669 | 3391.07 | 469.357 |
|  | Left Putamen | 3171 | 6106 | 4569.48 | 687.472 |
|  | Right Putamen | 3612 | 5606 | 4750.96 | 507.363 |
|  | Left Pallidum | 1189 | 2451 | 1778.44 | 279.913 |
|  | Right Pallidum | 1116 | 2378 | 1752.11 | 277.502 |
|  | Left Thalamus | 6358 | 9070 | 7487.85 | 694.711 |
|  | Right Thalamus | 6144 | 8448 | 7329.15 | 608.109 |
|  | Left Accumbens | 165 | 677 | 458.70 | 128.978 |
|  | Right Accumbens | 86 | 539 | 306.78 | 123.304 |
|  | Left Hippocampus | 2279 | 4397 | 3685.70 | 428.170 |
|  | Right Hippocampus | 2266 | 4631 | 3745.93 | 577.147 |
|  | Left Amygdala | 644 | 1729 | 1105.96 | 303.901 |
|  | Right Amygdala | 443 | 1984 | 1234.74 | 382.429 |

## All volumes are in native space in mm^3^. All volumes were created by FSL FIRST.
