# Supplementary figures and images for: Striatal and Hippocampal Atrophy in Idiopathic Parkinson’s Disease Patients without Dementia: A Morphometric Analysis
Source: Front Neurol. 2017 Apr 13;8:139. doi: 10.3389/fneur.2017.00139 (PMC5389981; doi:10.3389/fneur.2017.00139)

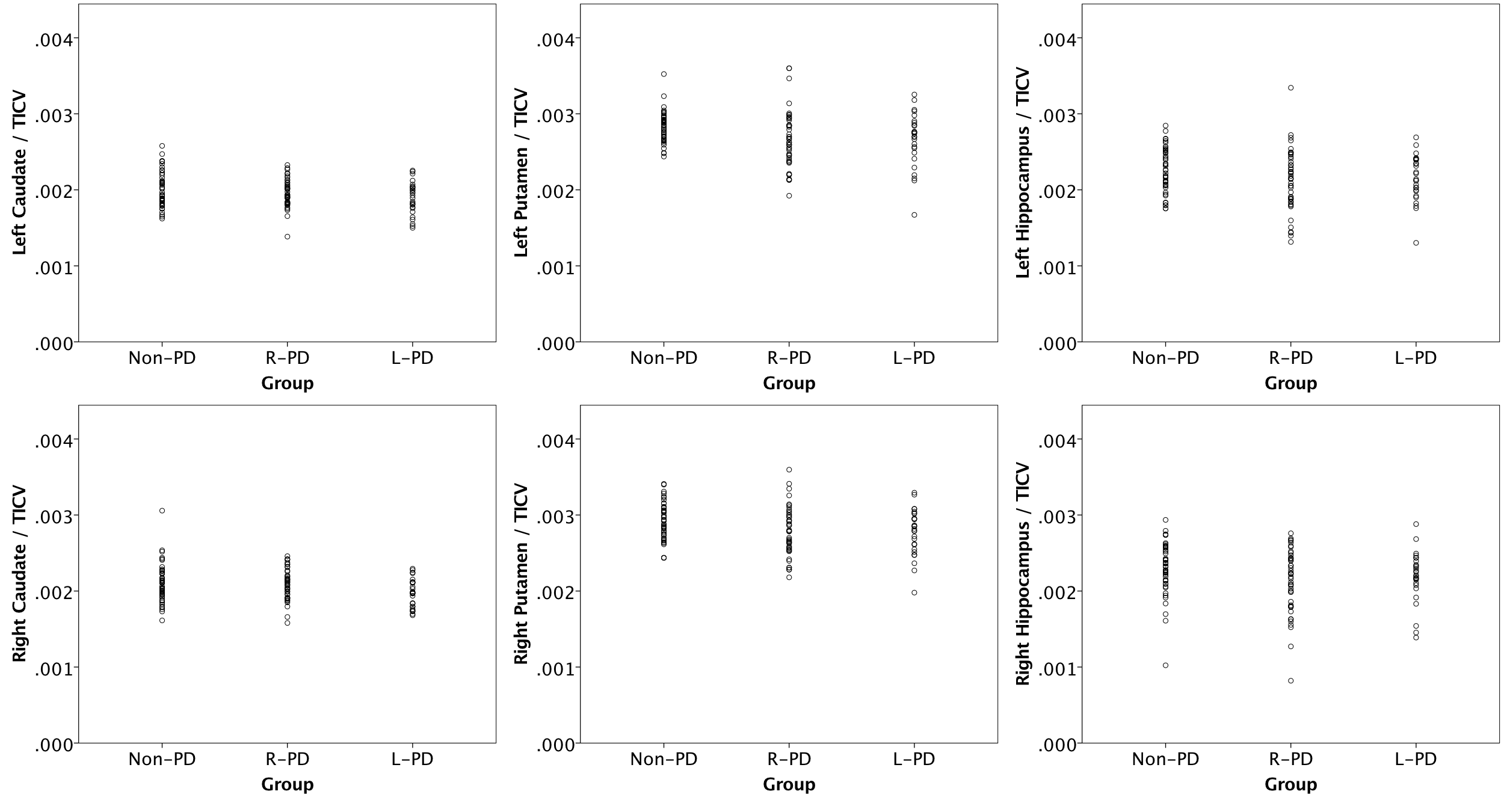

Supplement: Supplementary file 2 [file Image_1.TIFF]
